# Therapeutic expression of human clotting factors IX and X following adeno-associated viral vector–mediated intrauterine gene transfer in early-gestation fetal macaques

**DOI:** 10.1096/fj.201801391R

**Published:** 2018-12-05

**Authors:** Jerry K. Y. Chan, Irene Gil-Farina, Nuryanti Johana, Cecilia Rosales, Yi Wan Tan, Jessika Ceiler, Jenny Mcintosh, Bryan Ogden, Simon N. Waddington, Manfred Schmidt, Arijit Biswas, Mahesh Choolani, Amit C. Nathwani, Citra N. Z. Mattar

**Affiliations:** * Reproductive Medicine, KK Women’s and Children’s Hospital, Singapore, Singapore;; † Cancer and Stem Cell Biology Program, Duke–National University of Singapore (NUS) Medical School, Singapore; ‡ Department of Translational Oncology, German Cancer Research Center/National Center for Tumor Diseases, Heidelberg, Germany;; § University College London (UCL) Cancer Institute, University College London, London, United Kingdom;; ¶ Obstetrics and Gynaecology, Yong Loo Lin School of Medicine, National University of Singapore, Singapore;; ‖ SingHealth Experimental Medicine Centre, Singapore Health Services Pte, Singapore, Singapore;; # Institute for Women’s Health, University College London, London, United Kingdom;; ** Faculty of Health Sciences, Wits/South African Medical Research Council (SAMRC), Antiviral Gene Therapy Research Unit, University of the Witwatersrand, Johannesburg, South Africa; and; †† GeneWerk, Heidelberg, Germany

**Keywords:** nonhuman primate, immune tolerance, genotoxicity

## Abstract

Adeno-associated viral vectors (AAVs) achieve stable therapeutic expression without long-term toxicity in adults with hemophilia. To avert irreversible complications in congenital disorders producing early pathogenesis, safety and efficacy of AAV-intrauterine gene transfer (IUGT) requires assessment. We therefore performed IUGT of AAV5 or -8 with liver-specific promoter-1 encoding either human coagulation factors IX (hFIX) or X (hFX) into *Macaca fascicularis* fetuses at ∼0.4 gestation. The initial cohort received 1 × 10^12^ vector genomes (vgs) of AAV5-hFIX (*n* = 5; 0.45 × 10^13^ vg/kg birth weight), resulting in ∼3.0% hFIX at birth and 0.6–6.8% over 19–51 mo. The next cohort received 0.2–1 × 10^13^ vg boluses. AAV5-hFX animals (*n* = 3; 3.57 × 10^13^ vg/kg) expressed <1% at birth and 9.4–27.9% up to 42 mo. AAV8-hFIX recipients (*n* = 3; 2.56 × 10^13^ vg/kg) established 4.2–41.3% expression perinatally and 9.8–25.3% over 46 mo. Expression with AAV8-hFX (*n* = 6, 3.12 × 10^13^ vg/kg) increased from <1% perinatally to 9.8–13.4% >35 mo. Low expressers (<1%, *n* = 3) were postnatally challenged with 2 × 10^11^ vg/kg AAV5 resulting in 2.4–13.2% expression and demonstrating acquired tolerance. Linear amplification–mediated-PCR analysis demonstrated random integration of 57–88% of AAV sequences retrieved from hepatocytes with no events occurring in or near oncogenesis-associated genes. Thus, early-IUGT in macaques produces sustained curative expression related significantly to integrated AAV in the absence of clinical toxicity, supporting its therapeutic potential for early-onset monogenic disorders.—Chan, J. K. Y., Gil-Farina I., Johana, N., Rosales, C., Tan, Y. W., Ceiler, J., Mcintosh, J., Ogden, B., Waddington, S. N., Schmidt, M., Biswas, A., Choolani, M., Nathwani, A. C., Mattar, C. N. Z. Therapeutic expression of human clotting factors IX and X following adeno-associated viral vector–mediated intrauterine gene transfer in early-gestation fetal macaques.

Clinical gene therapy (GT) trials for severe monogenic diseases have demonstrated survival benefit over existing therapies. In recent phase I trials for hemophilia, adeno-associated viral vectors (AAVs)-GT achieved dose-dependent hematologic correction, with reassuring safety profiles (1, 2). Nevertheless, ongoing hemarthrosis in adults limits phenotype correction despite efficient transduction (3, 4). Early AAV-GT preceding permanent tissue damage is thus an attractive prospect for children with monogenic conditions (3–6). Infants with spinal muscular atrophy treated with self-complementary AAV (scAAV)9.CB.hSMN demonstrated significant dose-dependent neurologic improvement and higher unventilated survival when compared with controls (7). The most beneficial application of GT may be during early fetal development, particularly for syndromes that manifest *in utero* and in which postnatal therapeutic potential is limited by irreversible tissue damage or inefficient protein replacement (8–10), examples of which include neuronopathic Gaucher disease and other neurometabolic syndromes (11–13) and congenital factor X (hFX) deficiency, which causes perinatally lethal intracranial hemorrhage (14–16). In such conditions, better outcomes are demonstrated when genetic correction is attempted *in utero* rather than in later childhood, and improving accuracy of early prenatal testing is likely to drive the demand for fetal therapies (6, 17–22).

Clinical outcomes depend on fetal maturity status at intervention (23). Increased fetal stem cell receptivity to vector transduction (24–26), minimal pre-existing tissue damage (27, 28), and immune naïveté facilitating vector tolerance (20, 29–31) increase the likelihood of long-term phenotype correction. It is, however, critical to interrogate organ- and genotoxicity in addition to protein deficiency correction in GT applications—issues that have arisen in the successful gene transfer of young children with severe congenital immunodeficiency and spinal muscular atrophy (7, 32). Most intrauterine gene transfer (IUGT) animal models do not demonstrate the degree of transgenic protein production the we have described in nonhuman primates (NHPs), here and previously (29, 33). Murine IUGT with AAV1-human factor IV (hFIX) or AAV2-hFIX at 10^12^–10^13^ vector genomes (vgs)/kg showed persistent low transgene levels, requiring AAV readministration for therapeutic expression (31). Ovine AAV-IUGT produced only low expression up to 6 mo after peak expression at ∼3 wk after injection (34). NHP-IUGT with scAAV5- liver-specific promoter (LP)-1-hFVII maintained ∼20% expression at birth, successfully boosted, after loss of expression, by the alternate AAV8 serotype, which thereafter supported therapeutic FVII levels without a sustained immune response (35). Despite the expanding applicability of recombinant AAV pseudotypes engineered for improved safety *via* specific organ targeting and reduced insertional oncogenesis, a major limitation is its predominantly episomal nature (36–42). These animal studies demonstrate the progressive loss of expression expected after fetal or neonatal treatment as the recipient grows (43). Introduction of GT vectors during fetal development to induce immune tolerance and facilitate repeat AAV administration after birth (33, 44–46) can be a useful strategy for stabilizing waning protein expression in the hemophilias and neurometabolic disorders (11, 47). Despite the encouraging outcomes of clinical trials, high-dose AAV has resulted in complications associated with integration, direct cellular toxicity, and systemic inflammation, resulting in hepatocellular carcinoma in rodents, neurodegeneration in piglets and liver failure in juvenile NHPs (48, 49). Data regarding AAV-IUGT safety and efficacy can thus originate only from a high-fidelity NHP model in which longitudinal surveillance is clinically relevant (50). We have described long-term outcomes of liver-directed late-gestation IUGT in NHP by using the clinical vector scAAV-LP1-human codon-optimized human FIX transgene (FIXco) (5) in which a single injection at 0.9 gestation demonstrated therapeutic hFIX expression in most animals (29, 33). Low-expressing animals were safely boosted with only transient immune responses (33). Using hFX as a marker transgene in the same manner as hFIX, we interrogated the expression, immune response, and vector biodistribution after single-dose early-IUGT at ∼0.35–0.4 gestation in an established NHP model in which we used AAV5 and -8 vectors expressing either hFIX or hFX codon-optimized transgenes (50). This study is the first that we know of to describe in extensive detail the clinically relevant outcomes of early IUGT in a large number of NHPs. We report unexpectedly prolonged and stable postnatal transgenic protein production with only transient transaminitis, a high level of vector integration with no hotspots, and evidence of immune tolerance.

## MATERIALS AND METHODS

### Vector production

scAAV-LP1-hFIXco was produced as described by Nathwani *et al*. (44). scAAV-LP1-hFXco was produced similarly, replacing the hFIXco with the codon-optimized hFX transgene (hFXco), from pAV-LP1-hFX with a truncated poly-A. AAV-inverted terminal repeats with the LP1 promoter were cut with *Hin*dIII/*Mfe*I, ligated with T4 DNA ligase (New England Biolabs, Ipswich, MA, USA), and heat shocked into competent *Escherichia coli*, which were grown at 37°C overnight (51). Correct clones were identified after DNA extraction by digestion with *Hin*dIII/*Pst*I, *Ahd*I, and *Bgl*I. Plasmid expression of hFX expression was assessed by ELISA after transient transfection of HepG2 cells with Fugene (Roche, Basel, Switzerland), and clones were amplified with Megaprep (Qiagen, Limberg, The Netherlands). scAAV-LP1-hFIXco and scAAV-LP1-hFXco plasmids were used in adenovirus-free transient transfection of 293T cells to generate scAAV5 and scAAV8 pseudotypes, as described by Nakai *et al*. (45). Titers were determined by quantitative PCR with linearized plasmids used as standards.

### IUGT, fetal blood sampling, delivery and monitoring

All procedures were performed in *Macaca fascicularis* strictly adhering to recommendations from the Institutional Animal Care and Use Committee at the National University of Singapore and Singapore Health Services Pte, Ltd. (IACUC 2012/SHS/692). The *in vivo* work was conducted at the SingHealth Experimental Medicine Centre (SEMC, Singapore), accredited by the Association for Assessment and Accreditation of Laboratory Animal Care International. Timed mating and pregnancy monitoring are described in refs. 29 and 50. Vector was administered to macaques under ketamine sedation *via* intraperitoneal, intracardiac, or intravascular injection with an 18-gauge Quincke spinal needle (BD Biosciences, Franklin Lakes, NJ, USA) with ultrasound (US) guidance (**Table 1**). Injected volumes were restricted to 500 µl, and fetal heart rate was monitored for 5 min after injection. Routine maternal and fetal surveillance, fetal blood sampling (FBS) at ∼0.6 G, caesarean delivery, and infant hand-rearing were performed (50). From 1 yr of age, hepatic and other tissue biopsies were performed at 6-mo intervals to assess vector biodistribution and integration (29).

**TABLE 1:** Biodata and outcomes of fetuses injected in early gestation with scAAV-LP1-hFIXco and scAAV-LP1-hFXco

Infant	Gender	AAV	Gestation at IUGT (d)	Total dose (vg)	Dose in vg/kg	Route of delivery	Gestation at birth (d)	Birth weight (g)	Outcome (mo)
Factor IX									
e5001	Male	AAV5	0.35 (54)	1 × 10^12^	6 × 10^12^	IP	144	165	Stillborn*^a^*
e5002	Female	AAV5	0.36 (56)	1 × 10^12^	4.26 × 10^12^	IP	147	235	Alive (19)
e5003	Male	AAV5	0.37 (58)	1 × 10^12^	3.64 × 10^12^	IP	147	275	Alive (19)
e5004	Male	AAV5	0.45 (69)	1 × 10^12^	4.35 × 10^12^	IC	146	230	Deceased (1)*^b^*
e5005	Male	AAV5	0.46 (71)	1 × 10^12^	4.08 × 10^12^	IH	146	245	Alive (65)
e8001	Male	AAV8	0.46 (71)	0.2 × 10^13^	6.90 × 10^12^	IP	148	290	Alive (62)
e8002	Male	AAV8	0.61 (94)	1 × 10^13^	3.23 × 10^13^	IH	149	310	Alive (62)
e8003	Male	AAV8	0.48 (75)	1 × 10^13^	3.77 × 10^13^	IP	145	265	Alive (62)
Factor X									
e5001FX	Female	AAV5	0.48 (75)	1 × 10^13^	3.28 × 10^13^	IP	149	305	Alive (14)
e5002FX	Female	AAV5	0.37 (58)	1 × 10^13^	3.70 × 10^13^	IP	147	270	Alive (58)
e5004FX	Female	AAV5	0.47 (74)	1 × 10^13^	3.73 × 10^13^	IH	149	300	Deceased (15)*^c^*
e8001FX	Unknown	AAV8	0.31 (48)	1 × 10^13^	NA	IP	N.A.	N.A.	Miscarried
e8002FX	Unknown	AAV8	0.37 (57)	0.5 × 10^13^	NA	IP	N.A.	N.A.	Miscarried
e8003FX	Female	AAV8	0.37 (58)	1 × 10^13^	3.39 × 10^13^	IP	147	295	Alive (57)
e8004FX	Unknown	AAV8	0.38 (59)	1 × 10^13^	NA	IP	N.A.	N.A.	Miscarried
e8005FX	Female	AAV8	0.37 (57)	1 × 10^13^	2.74 × 10^13^	IP	149	365	Alive (52)*^a^*
e8006FX	Male	AAV8	0.35 (55)	1 × 10^13^	3.22 × 10^13^	IP	147	310	Alive (44)*^a^*

a FBS performed at 0.6 gestation. *^b^*Died of sepsis. *^c^*Unknown cause of death.

### Postnatal vector challenge

**Figure 1:**
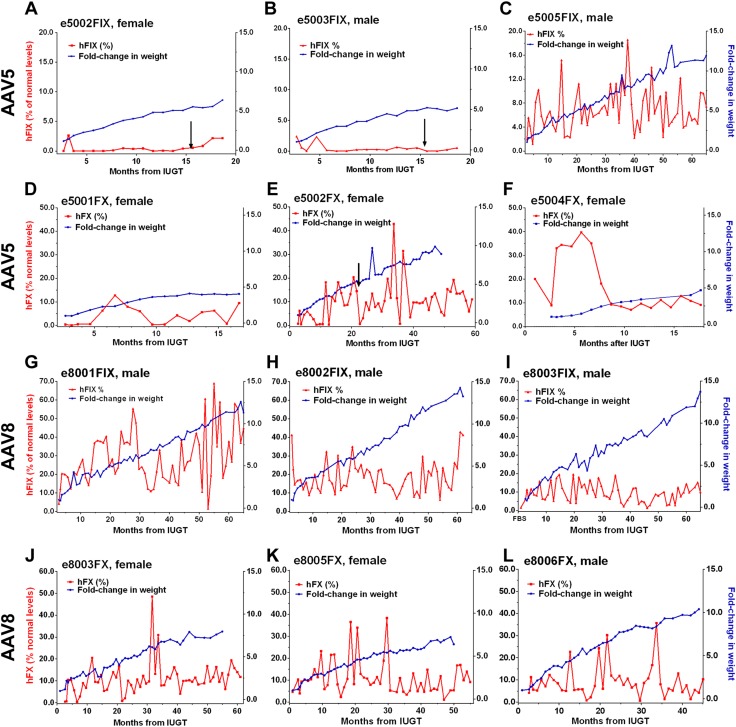
Individual hFIX and hFX expression in treated animals. *A*–*C*) Animals treated with AAV5-hFIX and monitored beyond infancy were e5002FIX (*A*) and e5003FIX (*B*), both of which showed subtherapeutic levels, and e5005FIX (*C*), which demonstrated therapeutic levels. (*D*–*F*) expression in e5001FX (*D*), e5002FX (*E*), and e5004FX (*F*) fluctuated between subtherapeutic (<1%) and high levels, whereas infant body mass steadily increased; e5004FX showed the more stable expression overall. *G*–*I*) AAV8 recipients were e8001FIX (*G*), e8002FIX (*H*), and e8003FIX (*I*), which sustained hFIX expression >1% (100% = 5 µg/ml) despite rapid growth. *J*–*L*) e8003FX (*J*), e8005FX (*K*), and e8006FX (*L*) fared similarly with median hFX expression in the therapeutic range (100% = 10 µg/ml). Arrows: postnatal VC.

Three infants with postnatal transgenic protein production of <1% were prepared for and serially monitored after vector challenge (VC), as previously described (33). Each received 2 × 10^11^ vg/kg body weight of AAV-hFIX or AAV-hFX and were monitored for immediate adverse reactions (**Fig. 1**). Treated subjects underwent open or percutaneous US-guided liver biopsies (Tru-Cut Needle; CareFusion, Basingstoke, United Kingdom) 3 mo after VC to assess vector load, hepatotoxicity, and vector integration. Wedge biopsies were collected *via* upper abdominal midline incision with hemostasis (50). All procedures were performed under ketamine sedation (IUGT, FBS, and VC) or general anesthesia (caesarean delivery, liver biopsy).

### Transgenic protein production

hFIX was analyzed by quantitative ELISA, as previously described (29). hFX was analyzed by using the same protocol with FX-EIA-C capture antibodies and FX-EIA-D detecting antibodies (Matched-Pair Antibody Set for ELISA of human factor X antigen, Affinity Biologicals, Ancaster, ON, Canada). Serum samples from injected offspring were diluted at 1:50 and 1:100 in HEPES-buffered saline-bovine serum albumin (BSA)-Tween 20 sample diluent for analysis. Human FX protein (ab62549; Abcam, Cambridge, United Kingdom) reconstituted in naive NHP serum and diluted serially from 0.075 to 10 µg/ml (0.75–100%) in HEPES-buffered saline-bovine serum albumin (BSA)-Tween 20 served as reference standards. Colorimetric change was developed and analyzed.

### Liver-specific expression

Liver-specific expression of hFIX and hFX was determined by RT-PCR, as previously described (29). The resultant cDNA (2 µl) was used to amplify a 617-bp region of the hFIX transgene with the forward 5′-TTTCCTGATGTGGACTATGT-3′ and reverse 5′-TCATGGAAGCCAGCACAGAACATG-3′ primers. The hFX transgene was amplified with the human-specific forward 5′-GAGAGGGGCGACAACAACCTCAC-3′ and reverse 5′-AGGCATCCTCCTGCTTGGTGTCG-3′ primers, producing a 568-bp amplicon. cDNA integrity was determined by amplifying a 604-bp region of the macaque β-actin gene by using forward 5′-TGACGGGGTCACCCACACTGTGCCCATCTA-3′ and reverse 5′-CTAGAAGCATTTGCGGTGGACGATGGAGGG-3′ primers. Cycling conditions were: 95°C for 5 min, 40 cycles of 95°C for 30 s, 57°C for 30 s (hFIX), or 60°C for 30 s (hFX, β-actin) and 72°C for 60 s, with final extension of 72°C for 7 min, with the product resolved on 2% agarose gel.

### Viremia and vector biodistribution

Assessment of viremia and vector biodistribution were performed as previously described for both hFIX and hFX-treated animals (29). In brief, 5 µl of cell-free maternal plasma or 15 µg genomic DNA extracted from tissue was amplified for an 84-bp region of the transgene encompassing the 3′ end of the codon-optimized transgene and the 5′ region of the SV40 late poly A sequence with 100 nM each of forward 5′-GGGCAAGTATGGCATCTACA-3′ and reverse 5′-AAAGCATCGAGTCAGGTCAG-3′ primers. Viral load was expressed as copies per microliter maternal plasma (viremia) or vector copy number (VCN) per diploid genome (6.6 pg of DNA); the calculated limit of detection was 1 vg per 455 diploid genomes. Equivalent loading was assessed by amplifying a 52-bp region of the macaque β-actin gene with forward 5′-TCCTGTGGCATCCACGAAA-3′ and reverse 5′-CCACGTCACACTTCATGATGG-3′ primers.

### Anti-AAV binding and neutralizing antibodies

Binding anti-capsid antibodies were assessed by sandwich ELISA, as previously described (29), using 5 × 10^8^ AAV8 or AAV5 particles as coating antigens, samples diluted 1:49 and goat anti-rhesus IgG (1:3999 dilution). Results were standardized to the negative baseline (readout of samples from unexposed macaques) and expressed as a ratio of the positive control (readout from a sensitized adult macaque strongly positive for anti-AAV8 or -5) (44). Neutralizing antibodies (NAbs) were assessed *in vitro* by incubating samples diluted 1:99 with 5 × 10^10^ AAV5 or -8 particles and then with 1 × 10^5^ 293T cells, as described in refs. 29 and 52. At least a 50% reduction in GFP transduction, relative to the control, was indicative of NAbs. Control subjects were immune-competent, unexposed, adult male macaques capable of producing anti-AAV antibodies as a primary response, which did not produce NAb during the first month after exposure.

### Cell-mediated immunity

Cell-mediated reactions to postnatal VC were analyzed by intracellular staining (ICS) adapted from a published protocol (53), as previously described (33). All antibodies and reagents were acquired from BD Biosciences unless stated otherwise. Briefly, frozen peripheral blood mononuclear cells (MNCs) were thawed, incubated overnight in Rosewell Park Memorial Institute (RPMI) 1640 with 10% fetal bovine serum and 1% penicillin/streptomycin at 37°C in 5% CO_2_, washed with HBSS supplemented with 2 U/ml DNase I, and resuspended in RPMI 1640. MNCs were first stimulated in the presence of either an AAV8 or -5 capsid peptide pool (final concentration, 2.5 μg per peptide per milliliter) in the presence of anti-CD28 (clone CD28.2), anti-CD49d (clone 9F10), and Brefeldin A. Nonviable and non-T cells were gated out with the Live/Dead Fixable Violet Dead Cell Stain Kit-Pacific Blue (Thermo Fisher Scientific, Waltham, MA, USA); anti-CD14-Pacific Blue (clone M5E2), anti-CD16-Pacific Blue (clone 3G8), and anti-CD20-Pacific Blue (clone 2H7; Bio-Rad, Oxford, United Kingdom); and T-cell subtypes were determined analyzed with anti-CD8-APC-H7 (clone SK1) and anti-CD4-Alexa700 (clone OKT4; eBioscience, San Diego, CA, USA); anti-CD95-PE-Cy5 (clone DX2) and anti-CD28-PE-Texas Red (clone CD28.2; Beckman Coulter, Brea, CA, USA); and anti-CCR7-PE (clone 150503; R&D Systems, Minneapolis, MN, USA) for 30 min at 4°C in the dark. After permeabilizing with Cytofix/Cytoperm for 20 min at room temperature, ICS was performed with anti-IFN-γ-APC (clone B27), anti-IL-2-FITC (clone MQ1-17H12), anti-TNF-α-PE-Cy7 (clone MAb11) and anti-CD3-PerCP-Cy5.5 for 30 min at 4°C in the dark. Fixed cells were analyzed by fluorescence-activated cell sorting with LSR Fortessa and FACSDiva software (BD Biosciences). Postacquisition analyses were performed with Summit 4.2 (Beckman Coulter). Single-color controls were added *via* Compbeads Anti-Mouse Ig, κ (BD Biosciences). Cells were gated onto CD3 and then onto CD8 or CD4 cells, which in turn were gated onto CD95 and CD28, to differentiate effector and memory cells, as previously described (33). Effector and memory cells were gated onto cytokines IL-2, IFN-γ, and TNF-α. Naive macaque MNCs incubated in phorbol 12-myristate 13-acetate and ionomycin (5 μg/ml) served as the positive control; the negative control consisted of MNCs incubated in peptide-free medium. Samples were read as positive if at least 0.05% of a subpopulation stained for a particular cytokine.

### Integration sites analyses by linear amplification–mediated-PCR and high-throughput sequencing

Linear-amplification–mediated (LAM) PCR was performed as described in refs. 29, 33, and 54. In brief, AAV flanking sequences from both AAV-hFIX and AAV-hFX vectors were amplified from 750 ng genomic DNA from liver biopsies by using the same primers (Supplemental Table S1). Restriction digest steps were performed with the *Mse*I and *Mlu*CI restriction enzymes (New England Biolabs). After 1 linear and 2 exponential PCRs, an additional PCR amplification with barcoded fusion primers was performed to prepare fragments for 250PE MiSeq sequencing (Illumina, San Diego, CA, USA). Raw sequences were analyzed by automated bioinformatics tools for quality filter, vector trimming, and identification of vgs [integration sites (ISs)] and vector-vector (concatemers) junctions. ISs were mapped to the macaque genome by using the University of California at Santa Cruz Blast-like alignment tool and were analyzed by automated data-mining tools to characterize the vector’s integration profile (55).

### Statistics

Results were analyzed using statistical software Prism v.6.04 (GraphPad Software, La Jolla, CA, USA). Data are shown as means ± sd, and analyzed using unpaired Student’s *t* tests. A value of *P* < 0.05 was considered significant. Frequency of integration sequence retrieval from tissue samples of subjects was compared by Fisher’s exact test.

## RESULTS

### Monitoring and survival of treated animals

Four groups of fetuses were treated: scAAV-LP1 serotypes 5 and 8, expressing either hFIX (5 µg/ml represents 100% normal levels) or hFX (10 µg/ml represents 100% normal levels) transgenes. Pregnant dams were assigned treatment groups based on anti-AAV5/8 seronegativity. AAV-hFIX IUGT (AAV5, *n* = 5; AAV8, *n* = 3) was performed at 48–94 gestational days (GDs) or 0.31–0.61 gestation (median, 0.37 G) (Table 1). Routes of injection were determined by fetal intrahepatic vein accessibility: 3 fetuses were injected intravenously or intracardially [using a technique similar to that employed in clinical fetal therapy of tachyarrhythmias (56)], and the remainder were injected intraperitoneally or intrahepatically. FBS was successfully performed in the e5001, which was stillborn at 144 GDs (0.9 G; Table 1). Fetal viability was confirmed by US. The first 5 fetuses (e5001, e5002, e5003, e5004, and e5005) received 1 × 10^12^ vg of AAV5-hFIXco to assess expression at a deliberately low starting dose and avoid potential toxic inflammatory reactions from vector overload. Interim analysis of e5001 by FBS (0.6 gestation) demonstrated hFIX levels of 1.7%. Higher doses of 0.2 × 10^13^ vg (*n* = 1) and 1 × 10^13^ vg (*n* = 2) of AAV8-hFIX were used thereafter to improve expression. AAV-FX animals received 1 × 10^13^ vg of scAAV-LP1-hFXco IP at 48-75GDs (0.31–0.48 gestation; AAV8, *n* = 6; AAV5, *n* = 3; Table 1). e8002FX received 0.5 × 10^13^ vg of available vector stocks (volumes restricted to 500 µl). FBS was performed at 83–99 GDs (0.54–0.64 gestation; *n* = 3). Animal loss from procedural technicalities occurred at a rate comparable to late IUGT (33). Infants were delivered at 144–149 GDs at median birth-weight of 275 g (range, 165–365 g) (50). They developed normally except for e5004, which perished from nosocomial pneumonia at 5 wk of age (Table 1).

### Dose-dependent transgenic protein production according to serotypes 5 and 8

**Figure 2:**
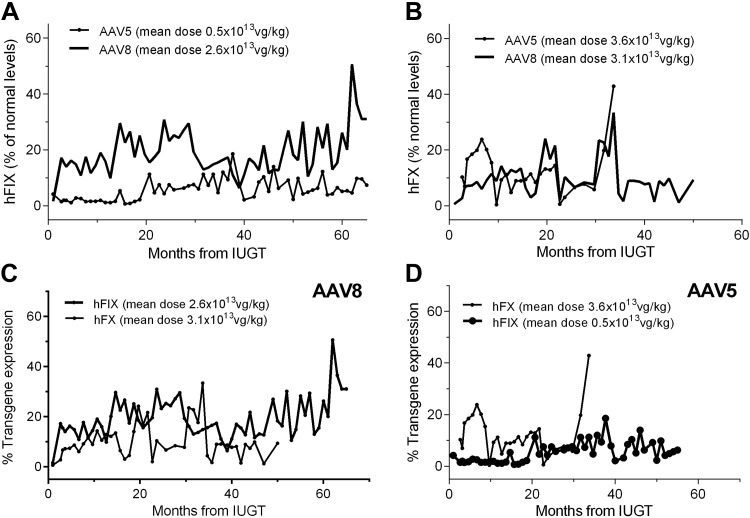
Serotype-dependent hFIX and hFX expression. *A*) AAV5 animals produced lower hFIX levels (5.6 ± 4.1% after 0.5 × 10^13^ vg/kg) than AAV8 infants (17.7 ± 5.7 after 2.6 × 10^13^ vg/kg). *B*) hFX expression was similar in AAV8 (9.8 ± 6.6%) and AAV5 animals (12.9 ± 10.3%) which received similar doses. *C*) AAV8 produced higher levels of hFIX (19.3 ± 8.0%) than hFX (9.8 ± 6.6%) at similar vector doses. *D*) AAV5 showed dose-dependent production of hFX (12.9 ± 10.3% after a dose of 3.6 × 10^13^ vg/kg) compared to hFIX (6.3 ± 4.1% after 0.5 × 10^13^ vg/kg).

AAV5-hFIX animals (1 female, 4 males) received a mean dose of 0.45 × 10^13^ vg/kg calculated by birth weight. Early hFIX expression ranged from 4.2% (e5001 at 0.6 gestation) to 3.0 ± 1.8% (e5001 at 0.9 gestation, stillbirth) and 4.9% (e5004, perimortem at 1 mo of age). Steady-state expression ranged from low levels of ∼0.6 ± 0.8% (e5002 and e5003) over ∼19 mo and 5–6-fold weight gain (Fig. 1*A, B*) to 6.8 ± 3.8% (e5005) over ∼51 mo and 10-fold weight gain (Fig. 1*C*; *P* < 0.0001). Both e5002 and e5003 expressed <1% of normal levels at 1 yr and underwent postnatal VC. AAV5-hFX animals (3 females) received a mean dose of 3.57 × 10^13^ vg/kg. hFX levels reached 0.4–0.7% at birth (e5001FX and e5002FX) and ranged from 27.9 ± 10.8% (∼9 mo, e5004FX) to 9.4 ± 10.0% (∼42 mo, e5002FX) after IUGT with weight gains of 4–8-fold (Fig. 1*D–F*). AAV8-hFIX–treated animals (3 males) receiving a mean dose of 2.56 × 10^13^ vg/kg showed expression of 1.6% at 0.6 gestation (30 d after IUGT, e8003) and between 4.2% (e8001) and 41.3% (e8002) at birth. Surveillance over ∼46 mo after IUGT demonstrated plateau levels from 9.8 ± 5.2% (e8003) to 25.3 ± 11.1% (e8001) with an 8–11-fold weight gain (Fig. 1*G–I*). hFX levels were <1% at 0.6 gestation (e8005FX and e8006FX by FBS) and at birth (e8003FX) among AAV8-hFX recipients given 3.12 × 10^13^ vg/kg. This level increased to steady-state mean levels between 9.8 ± 7.8% and 13.4 ± 10.3% over 31–35 mo, with a 6–9-fold weight gain (Fig. 1*J–L*). Mean hFIX levels from AAV5 animals that had received the lower vector dose of 0.5 × 10^13^ vg/kg (based on eventual birthweight), was lower over 65 mo than in AAV8 infants after 2.6 × 10^13^ vg/kg (5.6 ± 4.1% *vs.* 17.7 ± 5.7%; **Fig. 2*A***). hFX expression observed over 50 mo was similar between AAV8 and -5 recipients which had received doses of 3.1–3.6 × 10^13^ vg/kg (9.8 ± 6.6% *vs.* 12.9 ± 10.3%, Fig. 2*B*). In AAV8 animals receiving vector doses of 2.6–3.1 × 10^13^ vg/kg, higher levels of hFIX were observed than hFX (19.3 ± 8.0% *vs.* 9.8 ± 6.6%, Fig. 2*C*). AAV5 animals showed dose-dependent transgenic protein levels of 12.9 ± 10.3% hFX at a dose of 3.6 × 10^13^ vg/kg and 6.3 ± 4.1% hFIX after a dose of 0.5 × 10^13^ vg/kg (Fig. 2*D*).

### Immune responses to vector administration in IUGT recipients

**Figure 3:**
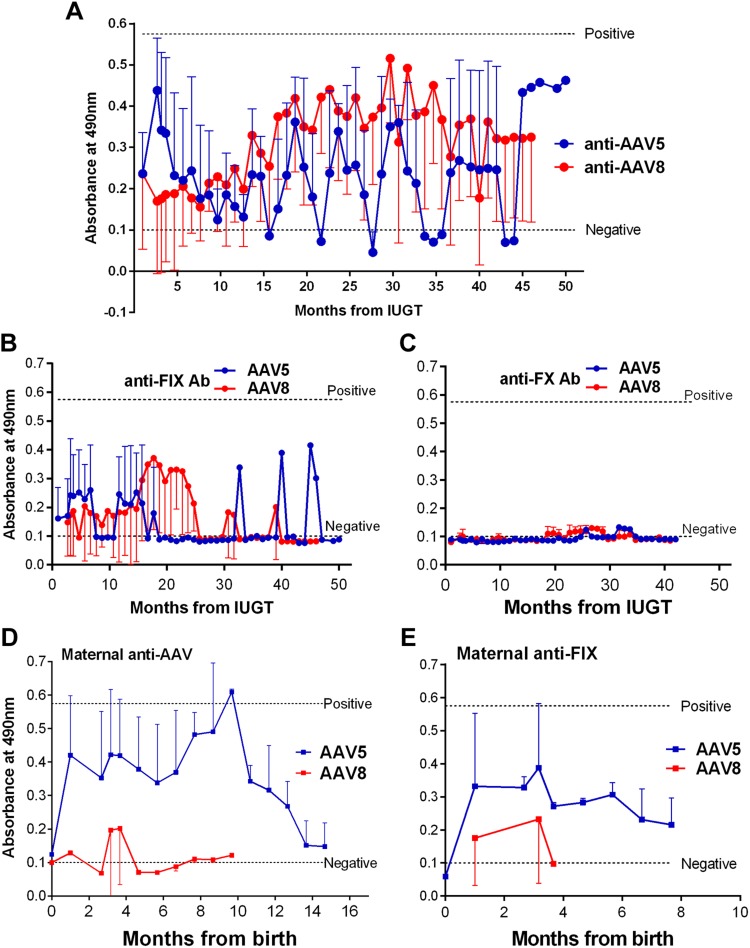
Humoral response to AAV capsid and transgenic protein. *A*) Anti-AAV binding antibodies remained below the positive threshold, and responses were similar to AAV8 and -5. *B*) Anti-hFIX antibodies remained in the negative range with no antibody production through a large part of the surveillance period. *C*) Anti-hFX antibody response remained negative. *D*, *E*) Maternal anti-AAV (*D*) and anti-transgene (*E*) responses were subdued overall.

Infants receiving either serotype demonstrated similar humoral responses to capsid and transgenic proteins. Persistent anti-capsid IgG antibodies were observed from 0.6 gestation throughout the surveillance period and remained below the positive threshold (**Fig. 3*A***). Responses to both transgenes were subdued (Fig. 3*B, C*). Maternal anti-AAV5 antibody expression was more robust than anti-AAV8 (Fig. 3*D*) and anti-transgene responses were mild (Fig. 3*E*) (29).

### Transgenic protein production and immune response after postnatal VC

**Figure 4:**
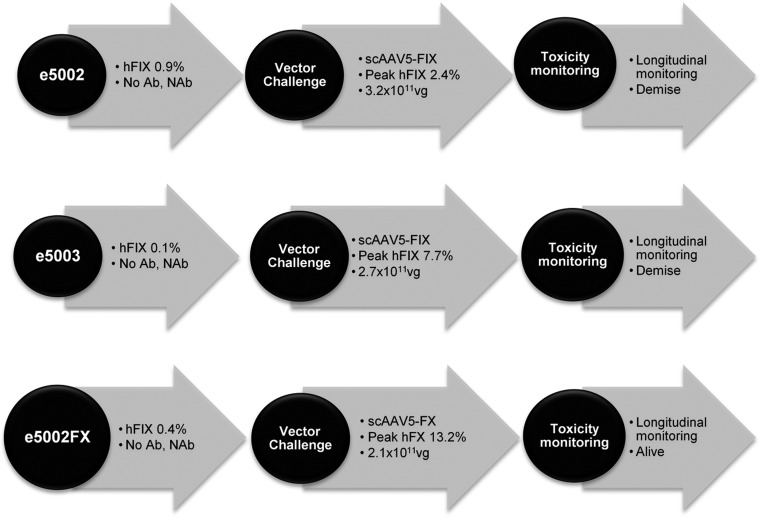
Vector challenge protocol. All subjects selected for postnatal VC were screened for anti-AAV binding (Ab) and NAbs underwent a prechallenge liver biopsy and were given 2 × 10^11^ vg/kg intravenously. All animals tolerated the additional dose of vector with no clinically overt immunologic responses. e5002 and e5003 died from anesthetic complications during the postchallenge liver biopsies.

**Figure 5:**
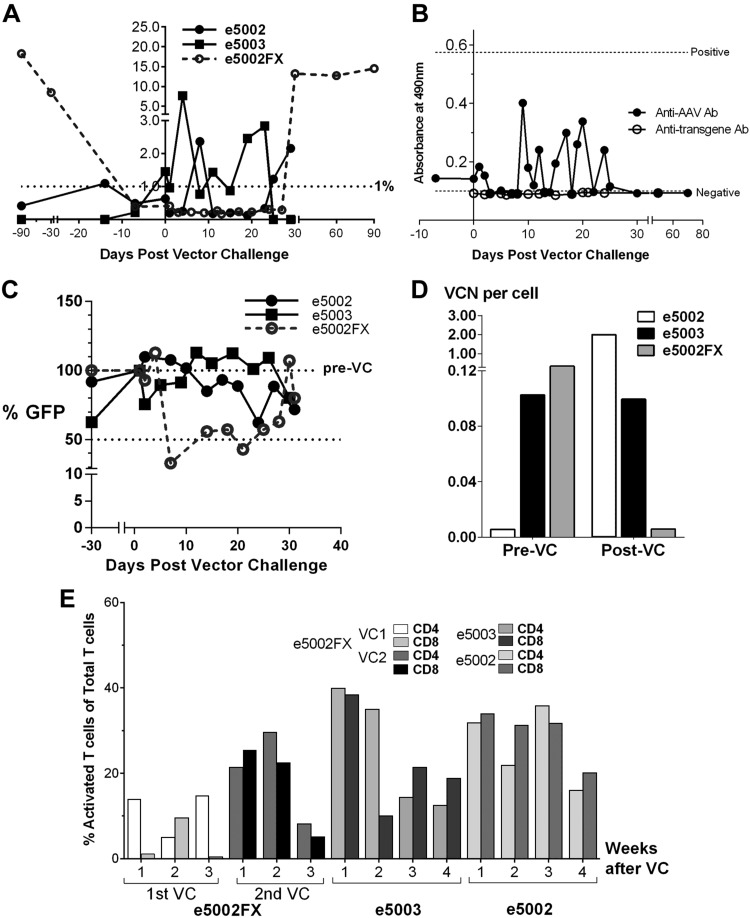
Postnatal VC and response. *A*) Three animals, e5002, e5003 (both AAV5-hFIX), and e5002FX (AAV5-hFX) received 2 × 10^11^ vg/kg AAV5. Transgene levels were <1% before VC (d 0). Brief improvements in expression were observed in e5003 after VC and were sustained over 23 d before being nullified. e5002 showed short spikes >1% at 8 and 24 d after VC, whereas e5002FX maintained <1% hFX until an increase was seen from d 27 after VC. *B*) Anti-AAV IgG antibodies increased mildly only in e5002 and e5003; no such reaction was observed in e5002FX, where anti-AAV5 IgG was undetectable. No IgG production against or was observed. *C*) The presence of NAbs was determined by loss of green fluorescent protein transduction to ≤50% of baseline transduction (normalized to 100%). e5002FX demonstrated NAbs from d 7 to 21 after VC before resolution. *D*) Pre- and postchallenge hepatic VCN showed various responses e5002 showed an increase from 0.01 to 2.00 copies/cell, whereas VCN in e5003 remained at 0.10 vector/cell. No further follow-up was possible, given that both recipients died at the postchallenge biopsy. A substantial decline from 0.35 to 0.01 copies/cell was observed in e5002FX. *E*) e5002FX showed an increase in activated CD4 and CD8 T cells producing TNF-α and IFN-γ from first to second postnatal VC, but this response quickly diminished by 3 wk after VC. Both e5002 and e5003 showed higher activated CD4 and CD8 T cells after VC, which similarly decreased over the following 4 wk.

e5002, e5003, and e5002FX were negative for binding and neutralizing anti-AAV antibodies and had normal transaminases, hematologic indices, and liver histology before VC (**Fig. 4**). None developed acute post-VC inflammatory responses over 90 d of monitoring. e5002 (3.2 × 10^11^ vg) showed transient hFIX elevation at 8 and 24 d after VC (peak, 2.4 ± 3.3%; overall mean, 0.8 ± 0.8%), whereas in e5003 (2.7 × 10^11^ vg) hFIX showed immediate improvement until ∼25 d (peak 7.7 ± 8.6%, overall 1.3 ± 2.0%). e5002FX (2.1 × 10^11^ vg) expressed <1% hFX until 27 d after VC, subsequently peaking at 13.2 ± 3.6% at 30 d (overall, 4.2 ± 6.4%). Transgene levels are summarized in **Fig. 5*A***. Anti-AAV IgG antibodies remained below the positive threshold in e5002 and e5003 and were largely undetectable in e5002FX (Fig. 5*B*). No IgG production against hFIX or hFX was observed. Only e5002FX produced NAbs from 7 to 21 d after VC, before a resolution that preceded the increase in hFX expression (Fig. 5*C*, VC at d 0). NAbs were not demonstrated in e5002 and e5003. e5002 showed a >300-fold increase in hepatic VCN (from 0.01 to 2.00 copies/cell), whereas the VCN in e5003 remained at 0.10 copies/cell. Hepatic VCN in e5002FX decreased from 0.4 to 0.006 copies/cell (Fig. 5*D*). Activated CD4 and CD8 T-cells expressing TNF-α and IFN-γ were observed against AAV5 capsid proteins (Fig. 5*E*). e5002FX showed increasing activated T cells from first to second postnatal VC, diminishing over the following 3 wk. Both e5002 and e5003 showed higher activated CD4 and CD8 T cells, decreasing over 4 wk. Both e5002 and e5003 perished from anesthesia-related complications following liver biopsies at 30 d after VC (Fig. 1).

### Biosafety: vector biodistribution and integration analysis

**Figure 6:**
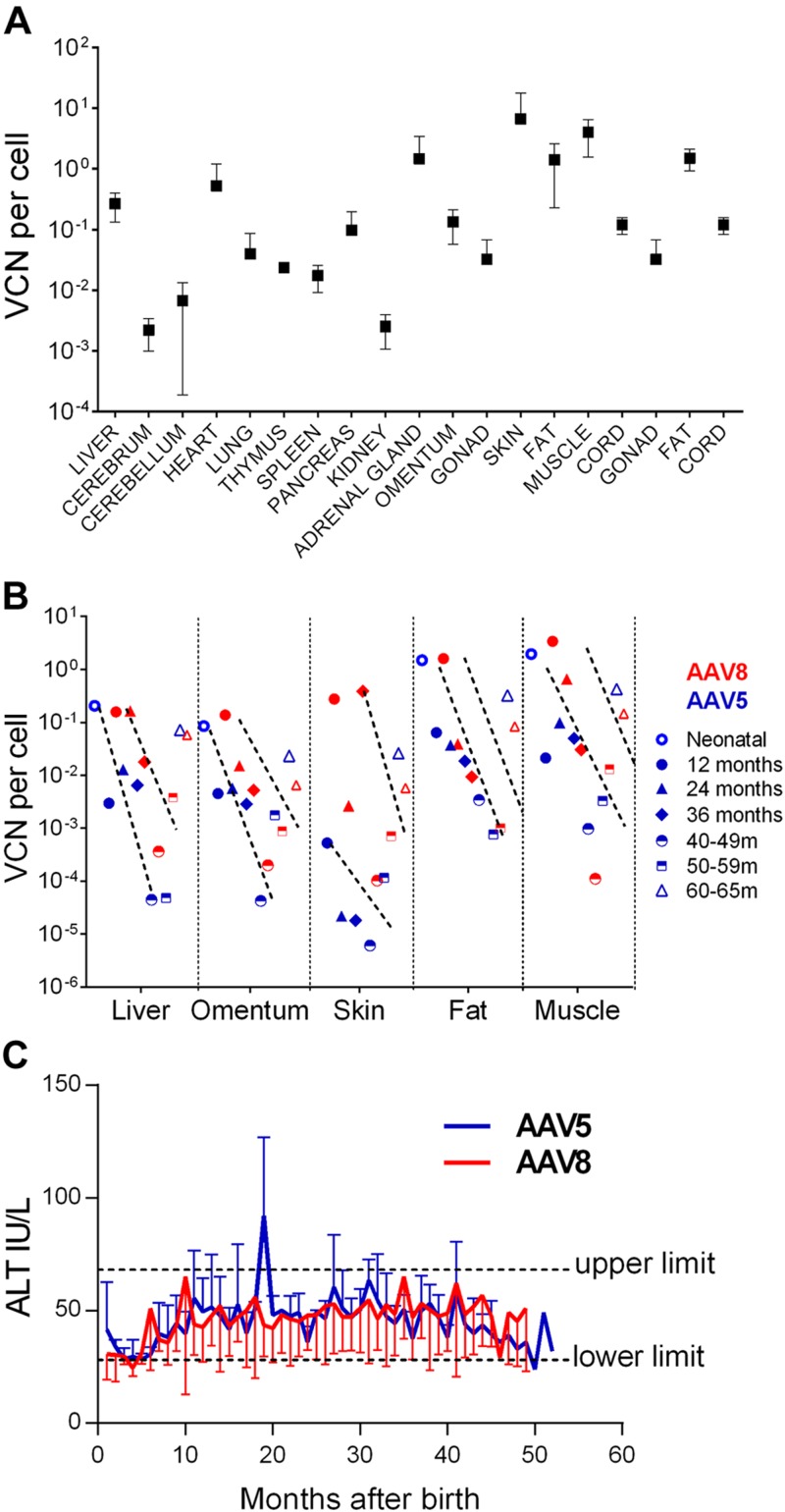
Vector biodistribution and hepatotoxicity. *A*) VCNs showed wide variation with most subjects expressing 1 vector copy every 10–1000 cells in the absence of specific organ tropism. *B*) Regardless of initial VCN, all tissues showed a substantial decline in tissue vector load of 1–3-log-fold every 12 mo; there was no difference between AAV5- and AAV8-treated animals. *C*) Liver-specific ALT levels increased intermittently, particularly in AAV8 recipients, but overall transaminases remained in the normal range.

Transduction was analyzed *via* serial biopsies of liver, omentum, skin, fat, and muscle from the neonatal period in e5001 (stillbirth, ∼80 d after IUGT), in e5004 at 5 wk (∼120 d), and from 12 to 42 mo in surviving offspring. Mean VCN ranged from 0.002 ± 0.001 copies/cell (cerebrum) to 6.7 ± 11.1 copies/cell (skin); hepatic VCN was 0.3 ± 0.1 copies/cell, and there was no demonstrable liver tropism (**Fig. 6*A***). We observed time-dependent log-fold decreases in VCN during each 12-mo period (1–36 m; *P* < 0.05). VCN in skin and muscle (highest starting vector load) decreased ∼2–3-log-fold by 42 mo (Fig. 6*B*). The decrease in VCN is shown in the context of sustained hFIX and hFX levels, as expression per hepatic VCN increased with time (Supplemental Fig. S1*A*). Liver-specific alanine transaminase (ALT) levels were generally normal, although it was increased at one time point in the AAV5 group (20 mo, 4–6 mo after VC; Fig. 6*C*). Aspartate transaminase levels were above the upper limit for the first 20 mo before decreasing; a trend toward higher levels was observed with AAV5 (Supplemental Fig. S1*B*). Despite this biochemical transaminitis, leukocyte counts and other hematologic indices registered only mild fluctuations within the upper normal limit, and there was no clinical evidence of hepatotoxicity (*e.g.*, anorexia, weight loss). Liver histology 3 mo before and after postnatal challenge did not demonstrate inflammatory cell infiltrates and showed normal hepatocellular architecture (Supplemental Fig. S1*C*, *D*).

**Figure 7:**
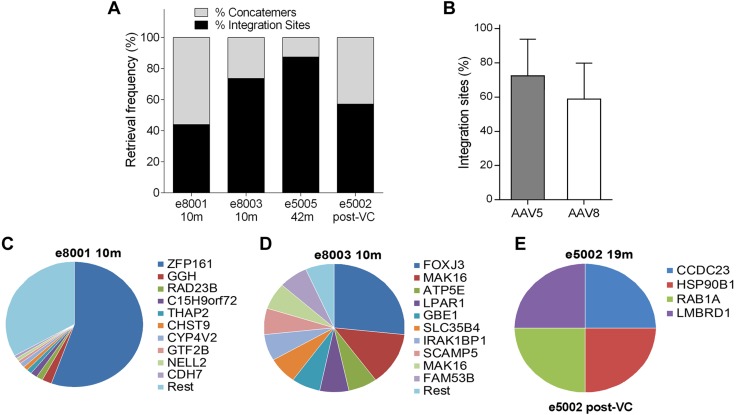
Vector ISs. *A*) Integration frequency increased from 40 to 70% at 10 mo to >80% at 42 mo in the different animals sampled. *B*) No differences in integration frequencies were noted between AAV5 and -8. *C*–*E*) Relative sequence counts of the 10 most prominent IS retrieved were calculated in relation to all uniquely mappable IS sequences in e8001 at 10 mo (*C*), e8003 at 10 mo (*D*, *E*), and e5002 at 19 mo (after VC). The RefSeq identities of the gene located adjacent to or at the IS are listed.

Our previous data in late-IUGT NHP demonstrated that integrated vector genome (∼10% of sequences at 2–10 mo after birth), rather than concatemers, accounted for the majority of vector persistence at later time points (87.4–100% at 18–49 mo after birth) (33). In the present study, LAM-PCR analyses revealed that 43.9–73.7% of vector sequences were integrated at 10 mo, increasing to 87.5% at 42 mo (**Fig. 7*A***). Amplicons obtained by LAM-PCR yielded 4,217,447 raw reads, enabling identification of 121 ISs corresponding to 111 unique and 14 multiple mappable ISs (**Table 2** and Fig. 7*A*). In e5002, the integrated vector accounted for 57.1% of retrieved sequences 3 mo after VC (19 mo of age). Integration frequencies were similar in both groups (72.3 ± 21.5% AAV5 *vs.* 58.8 ± 21.1% AAV8; *P* = 0.6; Fig. 7*B, C*). We performed semiquantitative estimation of the sample clonal size by analyzing the 10 most frequently retrieved clones from the different samples (Fig. 7*D–F*). Only two of these corresponded to integration events in or near cancer-related genes *RAD23B* and *RAB1A* (refer to the retroviral tagged cancer gene databases: the Cancer Genomics Consortium, *https://www.cancergenomics.org/*, and the cBio Portal for Cancer Genomics, *http://www.cbioportal.org/*), and none was retrieved at consecutive time points in the same animal or from different animals. Considering all integration events identified, 7.2% of integration events were located within or near cancer-related genes, which did not significantly differ from the 8.9% found in a synthetic random dataset. No significant targeting of chromosomes or gene coding regions were identified (Supplemental Fig. S1*E*, *F*), and there were no integration events retrieved from the genomic regions previously reported to be involved in AAV-associated oncogenesis (48).

**TABLE 2:** Vector ISs retrieved from liver biopsies

Animal ID	Gender	Months after birth	Raw reads	IS reads	Total IS	Unique IS	Multiple IS	IS/μg DNA
e5002	Female	19*^a^*	1,192,771	4	4	4	4	5.33
e5005	Male	42	194,372	7	1	-	1	1.33
e8001	Male	10	1,077,767	262	99	96	3	132
e8003	Male	10	1,752,537	28	17	11	6	22.67

Multiple IS, multiple mappable ISs; unique IS, uniquely mappable ISs. *^a^*Animal subjected

to postnatal VC 16 mo after birth.

## DISCUSSION

Recent AAV-GT hemophilia trials have demonstrated marked improvements in hemostatic stability and significantly reduced factor use in adults (3, 4, 57). Our data affirm the ability of early AAV-IUGT to generate curative expression of hFX and hFIX, stable for >4 yr, despite a several-fold weight increase (signifying hepatic growth), primarily represented by highly divergent and genome-wide vector integration within genes and introns in equal measure without demonstrable hotspots. No conclusions can be drawn as to the impact of administration route, as the numbers are too small for comparison. We anticipate that the intravenous route is the most efficient (58), but it results in longer procedures, potentially greater fetal stress or injury, and more prolonged exposure to anesthesia than intraperitoneal injections (unpublished results). Administration and surveillance strategies used for early and late IUGT were intentionally similar, as all procedures are clinically applicable and resulted in therapeutic expression levels for hemophilia B. Although the hemophilias are not conditions that require *in utero* rescue from the perspective of disease onset, it is encouraging that liver-directed AAV-driven GT can be performed at 0.4 gestation with a reasonable expectation of efficacy, safety, and technical feasibility. Substituting the relevant transgene for an inborn error of metabolism or other similar condition into AAV5 or -8 can be expected to effectively rescue a disease causing early fetal damage, the most cogent argument for early IUGT.

Our data suggest that vector integration has a similarly substantial and durable influence on stable long-term expression after both early and late IUGT, considering the low percentage of remaining episomes in both 0.4 and 0.9 gestation animals by 42–47 mo (33). It is not possible to conclude at the present time whether this finding represents a translational advantage or risk in the longer term, although the biosafety data would suggest low overall integration-related risk. Our previous work showed that AAV8 is the more efficient pseudotype for liver-directed GT in 0.9 gestation fetal and adult macaques (29, 33, 59). However, with IUGT at 0.4 gestation, AAV8 and -5 demonstrate equivalence in their efficacies as seen by their comparable hFX expression; lower expression among AAV5-hFIX recipients was largely related to the log-fold lower dose used, given that the number of males was similar in both groups. This finding demonstrates the utility of both AAV8 and -5 for liver-directed early-IUGT strategies for a range of congenital monogenic conditions with optimized transgenes. From comparative studies of AAV8-GT in infant and juvenile rodents and NHPs, clear correlation is observed between age at treatment and hepatocyte transducibility: earlier AAV8-GT produces higher, but ultimately more unstable, transduction caused by rapid liver growth and episomal loss within 1–3 mo of vector delivery, with expression typically reaching a plateau (60, 61). Hepatocyte transduction is also dose- and serotype-dependent, as demonstrated by greater expression with AAV8 (90–100% transduction) than with AAV2 (5–10% transduction) (62–64) and the linear dose-response relationship observed in adult macaques given 10^10^–10^12^ vg/kg of AAV8-hFIX (59).

Given these observations we had anticipated greater transgenic protein production with early-IUGT than was eventually achieved. The initial supraphysiological expression observed at 0.9 gestation was not seen at 0.4 gestation, which probably reflects superior episomal loss from rapid intrauterine growth of early-IUGT recipients, although median birth weights were comparable in both early and late-IUGT cohorts [275 *vs.* 267.5 g (range 205–290 g)] (33). In NHP pregnancies harvested at 0.5 gestation (median, 77.5 GDs; range; 71–83 d; *n* = 10), the median fetal weight was 46.0 g (36.2–72.0 g) and livers weighed ∼1.9 g (1.6–4.1 g; unpublished results). Using this fetal weight as reference, the concentration of vector achieved with 1 × 10^13^ vg was 2.2 × 10^14^ vg/kg, 1-log-fold higher than the 1.6 × 10^13^ vg/kg dose given to the late-IUGT recipients (29, 33). At 46 g at IUGT, the injected fetus is assumed to contain ∼5 × 10^10^ cells (65); at the median birth weight of 275 g the total number of cells per infant is expected to reach ∼3 × 10^11^ cells, over a mean of 60 d. Late-IUGT animals grew 2–3-fold over the first 60 d of life (injections were performed 1 wk before birth). With 1-log-fold higher vector load at early-IUGT to 0.6-log-fold increase in cell numbers, it is anticipated that, given the same efficacy of transduction, early-IUGT recipients would have higher expression from greater cellular transduction. We instead have observed lower expression, and posit that gestation-sensitive differences in the metabolic state of hepatocytes may be important contributory factors (63). Possible saturation kinetics were seen with AAV5-hFIX and AAV5-hFX, as there was no discernible improvement in transgenic protein production with log-fold increases of vector. The reason remains unknown, but we postulate that the early fetal liver is more resistant to AAV5 transduction, perhaps because of cell surface receptors or other dynamics of vector-cell interactions. In addition, it is known that hFIX increases steadily in childhood, whereas hFX levels are significantly lower at birth, only reaching adult levels by 5 yr of age (66). Initially low transgenic protein levels reflect hepatic functional immaturity at the time of AAV-GT and eventually rise to clinically normal levels with age. The differences in individual transgenic protein production from AAV8 and -5 are not clinically relevant here, given that steady-state expression of both transgenes reached therapeutic levels.

Immune responses to the primary AAV dose remained low, and the distinct immunogenicity of AAV5 observed among immune-mature recipients (late-gestation fetuses and adults) was absent in these early recipients (33, 59). Although T-cell assays were not performed after early-IUGT, sustained expression in most recipients supports the absence of a primary cellular immune response. A transient increase in ALT in AAV5 animals was not a direct result of the postnatal challenge (occurring 4–6 mo after VC), and this biochemical observation did not coincide with loss of transgenic protein production in the surviving AAV5-treated animals. Maternal IgG production was observed during the first 12 mo with AAV5 demonstrating greater immunogenicity than AAV8, consistent with our prior observations in late-gestation IUGT (29, 33). AAV5-injected dams had 1.5–1.8-fold higher anti-capsid and anti-hFIX antibody levels compared to offspring (similar levels with AAV8). It is possible that antibodies detected in offspring are transplacentally trafficked maternal antibodies, which may have accounted for some of the humoral response in treated infants. It is also possible that prolonged low-level anti-AAV IgG in early recipients indicates a mild primary response in most animals. Transplacental maternal immune influence on fetal expression has been described in murine experiments (67, 68). The degree to which transgenic protein production is influenced by maternal immunity is unknown; this may be a contributing factor toward low expression and warrants further investigation.

Post-VC responses in 0.4 gestation animals (compared to 0.9 gestation) demonstrated similar transgenic protein production, lower anti-AAV IgG production (remaining well below the positive limit), transient NAb expression in 1 of 3 challenged animals *vs.* NAb following all 3 challenges after late-IUGT, and a higher percentage of activated T cells expressing intracellular cytokines (33). Early NAb and activated T-cell production may have prevented transduction in e5002FX *via* immune-mediated clearance of transduced cells or prevention of vector entry. Recovery of expression in e5002 and e5002FX could be attributed to the eventual loss of T cells and NAb. Given this immune reaction, even the brief appearance of NAb can influence the outcomes of postnatal boosting. Although it is generally believed that early-gestation fetuses are immune naive, our data suggest that they are capable of immune responses and retain partial ability to eliminate transduced hepatocytes. Despite this, sustained transgenic protein production during this period suggests a certain degree of peripheral tolerance that facilitates postnatal boosting. A higher VC dose may overcome this barrier and remains to be tested.

Our data revealed no adverse events derived from direct AAV toxicity or insertional mutagenesis during the 4-yr surveillance in contrast to recent studies that used log-fold higher doses (49, 69). The sustained high transgenic protein production with rapidly decreasing VCN suggests that long-term transgene production derives from integrated vector more than from concatemers, although we did not demonstrate this with certainty; we also did not observe evidence of liver cancer.

The number of animals with long-term follow-up was small, ultimately limiting the power of important conclusions regarding durability of expression and immune response. Pregnancy losses were expected, given the high incidence among captive-bred NHPs (29, 50). There was no increase in postnatal mortality compared with our late-IUGT cohort apart from procedure-related deaths and nosocomial infections. The ratio of females to males was higher in the AAV5 group, but lower in the AAV8 group, and this would have influenced transgenic protein production readouts to some extent, given the influence of gender, as we have previously described (33). We did not determine the dose-response to AAV5-FIX because of experimental constraints imposed by the real-time availability of pregnant animals. The fetuses of AAV5-seronegative mothers were the first to be treated when there was a primary need to establish procedural safety. The initial lower dose of 1 × 10^12^ vg/fetus was based on previous studies that determined the effective dose of this vector in mice and adult macaques sufficient to achieve therapeutic expression (44, 70). The administered dose was further constrained by the volume limitation imposed by fetal size and was deliberately kept low to avoid circulatory embarrassment. Fetuses were subsequently treated with a higher dose of AAV8-hFIX when the suboptimal therapeutic achievements of AAV5-hFIX recipients became evident. We performed ICS for cytokines in activated T cells rather than enzyme-linked immunospot (ELISPOT). We chose this method because of the advantage of simultaneous assessment of multiple surface markers and effector cytokines reflecting the phenotype and function of reactive T cells. Although there are advantages to ELISPOT, we preferred the range and quantitative readouts of flow cytometry (71). Although the method may be nonstandard compared to ELISPOT, there is a growing number of studies applying ICS as the method of choice because of the greater flexibility and breadth of this assay. In NAb assays we did not perform serial dilutions, and although these semiquantitative assays suggest that high titers of NAb were not present, we cannot exclude the presence of low NAb titers in these samples.

In summary, early-IUGT can make a critical contribution in the therapeutic arsenal for congenital diseases that produce pathology in early development that may otherwise result in fetal loss or severe permanent handicap. Within the parameters of this research, we have demonstrated its usefulness in achieving curative levels of transgenic protein. Compared with late-IUGT, potential benefits extend from correction of early fetal pathogenesis to a higher degree of immune tolerance to postnatal repeat treatment to boost suboptimal outcomes. It can be interpreted from this study that serotype, dose, production of NAb and a stimulated T-cell response are factors critical to a long-term approach. Further immune modulation may still be necessary, as practiced in recent AAV clinical trials (3, 4, 72). No adverse effects of AAV toxicity or integration were observed during 4 yr of surveillance. Long-term therapeutic expression relies significantly on an integrated vector. Individual therapeutic strategies should be optimized further in the NHP model to assess translational potential.

## Supplementary Material

This article includes supplemental data. Please visit *http://www.fasebj.org* to obtain this information.

## Abbreviations

AAV: adeno-associated viral vector
ALT: alanine transaminase
ELISPOT: enzyme-linked immunospot
FBS: fetal blood sampling
FIXco: codon-optimized factor
FXco: codon-optimized human FX transgene
GD: gestational day
GT: gene therapy
ICS: intracellular staining
IS: integration site
IUGT: intrauterine gene transfer
LAM: linear amplification–mediated
LP: liver-specific promoter
MNC: mononuclear cell
NAb: neutralized antibody
NHP: nonhuman primate
scAAV: self-complementary AAV
US: ultrasound
VC: vector challenge
VCN: vector copy number
vg: vector genome
