# Supplementary figures and images for: Therapeutic expression of human clotting factors IX and X following adeno-associated viral vector–mediated intrauterine gene transfer in early-gestation fetal macaques
Source: FASEB J. 2018 Dec 5;33(3):3954–67. doi: 10.1096/fj.201801391R (PMC6404563; doi:10.1096/fj.201801391R)

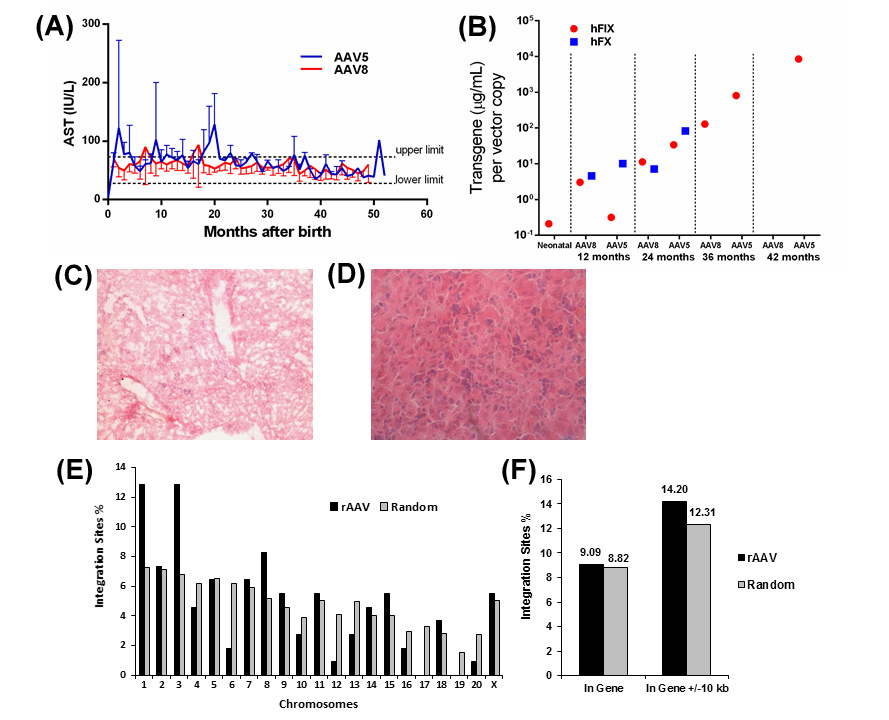

Supplement: Supplementary file 1 [file fj.201801391R.sf1.jpg]
