# Supplementary material for: Therapeutic expression of human clotting factors IX and X following adeno-associated viral vector–mediated intrauterine gene transfer in early-gestation fetal macaques
Source: FASEB J. 2018 Dec 5;33(3):3954–67. doi: 10.1096/fj.201801391R (PMC6404563; doi:10.1096/fj.201801391R)
Supplement: Supplementary file 2 [file fj.201801391R.sd1.docx]

**Supplemental Table 1.**

**AAV peptide sequences**

| **AAV8 peptide sequences for ICS** | **AAV5 peptide sequences for ICS** |
| --- | --- |
| H-AGGGAPMADNNEGAD-OH | H-QGADGVGNASGDWHC-OH |
| H-PMADNNEGADGVGSS-OH | H-VGNASGDWHCDSTWM-OH |
| H-NEGADGVGSSSGNWH-OH | H-GDWHCDSTWMGDRVV-OH |
| H-GVGSSSGNWHCDSTW-OH | H-DSTWMGDRVVTKSTR-OH |
| H-SGNWHCDSTWLGDRV-OH | H-GDRVVTKSTRTWVLP-OH |
| H-CDSTWLGDRVITTST-OH | H-TKSTRTWVLPSYNNH-OH |
| H-PQYGYLTLNNGSQAV-OH | H-NFEFTYNFEEVPFHS-OH |
| H-LTLNNGSQAVGRSSF-OH | H-YNFEEVPFHSSFAPS-OH |
| H-GSQAVGRSSFYCLEY-OH | H-VPFHSSFAPSQNLFK-OH |
| H-GRSSFYCLEYFPSQM-OH | H-SFAPSQNLFKLANPL-OH |
| H-YCLEYFPSQMLRTGN-OH | H-QNLFKLANPLVDQYL-OH |
| H-FPSQMLRTGNNFQFT-OH | H-LANPLVDQYLYRFVS-OH |
| H-RNSLANPGIAMATHK-OH | H-PQPNGMTNNLQGSNT-OH |
| H-NPGIAMATHKDDEER-OH | H-MTNNLQGSNTYALEN-OH |
| H-MATHKDDEERFFPSN-OH | H-QGSNTYALENTMIFN-OH |
| H-DDEERFFPSNGILIF-OH | H-YALENTMIFNSQPAN-OH |
| H-FFPSNGILIFGKQNA-OH | H-TMIFNSQPANPGTTA-OH |
| H-GILIFGKQNAARDNA-OH | H-SQPANPGTTATYLEG-OH |
| H-MLTSEEEIKTTNPVA-OH | H-QYSTGQVTVEMEWEL-OH |
| H-EEIKTTNPVATEEYG-OH | H-QVTVEMEWELKKENS-OH |
| H-TNPVATEEYGIVADN-OH | H-MEWELKKENSKRWNP-OH |
| H-TEEYGIVADNLQQQN-OH | H-KKENSKRWNPEIQYT-OH |
| H-IVADNLQQQNTAPQI-OH | H-KRWNPEIQYTNNYND-OH |
| H-LQQQNTAPQIGTVNS-OH | H-EIQYTNNYNDPQFVD-OH |

**SUPPLEMENTAL FIGURE S1 LEGEND**

(A) By comparing VCN among recipients of either serotype with the respective transgenic protein production at the same time-points, transduction efficacy was assessed by expressing hFIX per hepatic vector copy. From the two deceased AAV5 recipients transduction efficacy was 0.2µg/mL of hFIX per vector copy within the first postnatal month. At 12 months, mean expression from AAV8 was 3.1µg/mL hFIX and 4.6µg/mL hFX per vector copy, while expression from AAV5 was 0.3µg/mL hFIX and 10.1µg/mL hFX per vector copy. At 24 months median expression from AAV8 was 11.3µg/mL hFIX and 7.1µg/mL hFX per vector copy, and 33.9µg/mL hFIX and 83.1µg/mL hFX from AAV5. Beyond 36 months VCN in both groups of recipients were very low, and most still maintained adequate transgenic protein production, thus expression from AAV8 was 129.7µg/mL per vector copy at 36 months. AAV5 expression ranged from 806.7 µg/mL at 36 months to 8510.3 µg/mL at 42 months per vector copy. No differences were noted between the transduction efficacies of each serotype at the different intervals regardless of the transgene expressed. (B) Mean aspartate transaminase (AST) levels were above the upper limit for the first 20 months before a general decrease was observed; a trend towards higher AST was observed with AAV5. Pre-vector challenge hepatic wedge biopsies stained with H&E and viewed at (C) 10x and (D) 40x showing normal architecture and cell morphology (representative liver histology from e5003). (E) Chromosomal distribution of retrieved IS was analysed and compared to a synthetic random dataset of 8,628 IS to determine eventual integration hotspots. (F) Distribution of the IS within gene coding and nearby regions in comparison to a random dataset.
